# Stem cell differentiation increases membrane-actin adhesion regulating cell blebability, migration and mechanics

**DOI:** 10.1038/srep07307

**Published:** 2014-12-04

**Authors:** Kristina Sliogeryte, Stephen D. Thorpe, David A. Lee, Lorenzo Botto, Martin M. Knight

**Affiliations:** 1 Institute of Bioengineering and School of Engineering and Materials Science, Queen Mary University of London, Mile End Rd, London, E1 4NS, United Kingdom

## Abstract

This study examines how differentiation of human mesenchymal stem cells regulates the interaction between the cell membrane and the actin cortex controlling cell behavior. Micropipette aspiration was used to measure the pressure required for membrane-cortex detachment which increased from 0.15 kPa in stem cells to 0.71 kPa following chondrogenic differentiation. This effect was associated with reduced susceptibility to mechanical and osmotic bleb formation, reduced migration and an increase in cell modulus. Theoretical modelling of bleb formation demonstrated that the increased stiffness of differentiated cells was due to the increased membrane-cortex adhesion. Differentiated cells exhibited greater F-actin density and slower actin remodelling. Differentiated cells also expressed greater levels of the membrane-cortex ezrin, radixin, moeisin (ERM) linker proteins which was responsible for the reduced blebability, as confirmed by transfection of stem cells with dominant active ezrin-T567D-GFP. This study demonstrates that stem cells have an inherently weak membrane-cortex adhesion which increases blebability thereby regulating cell migration and stiffness.

Mesenchymal stem cells exhibit inherent plasticity in terms of their ability to differentiate into different lineages including osteoblasts, chondrocytes, adipocytes and neuron like cells. Human mesenchymal stem cells (hMSCs) are softer than differentiated cells^1^ which is likely to influence cellular functions including mechanotransduction and migration. Previous studies have examined the role of nucleus biomechanics and changes in chromatin condensation in this biomechanical phenomenon^2^. The present study investigates the interaction between the cell membrane and the actin cortex. In particular we examine the role of ERM proteins and how these regulate cell mechanics and membrane bleb formation during chondrogenic differentiation.

In eukaryotic cells, the lipid membrane is connected to the actin cortex via the family of ERM linker proteins, including ezrin, radixin and moesin^3^. Localised breakdown of the cortical cytoskeleton or detachment of the membrane from the cortex following rupture of these linker proteins, results in the formation of a membrane bleb. The bleb expands due to cytoplasmic pressure until polymerisation of actin beneath the membrane slows bleb growth and may eventually cause bleb retraction^4^,^5^,^6^. Thus blebs are different from other cellular protrusions, such as filopodia or lamellipodia where the membrane is pushed forward by actin filament polymerisation^7^. Bleb formation is known to occur during apoptosis^8^, but is also observed in healthy cells during cytokinesis^9^, spreading^10^ and migration^11^. Although non-apoptotic blebbing has been reported in stem cells^12^, no previous studies have examined the biomechanics of stem cell bleb formation. The aim of this study was therefore to quantity membrane-actin adhesion and to investigate how this changes with differentiation, leading to alterations in cellular mechanics and susceptibility to bleb formation.

Here we utilise a combined experimental and computational approach based on micropipette aspiration. We show that hMSCs have lower bond strength between the cell membrane and the cortical actin compared to differentiated cells and that this increases the susceptibility to membrane blebbing leading to lower cell stiffness. We then show that the lower bond strength in hMSCs is associated with lower expression of the ERM linker protein, ezrin, as well as changes in actin organisation and dynamics. Finally we show that overexpression of ezrin increases the mechanical properties of hMSCs replicating the mechanical behaviour observed in differentiated cells. This demonstrates that the weaker ERM-dependent membrane-cortex interaction in hMSCs, increases bleb formation and cell deformability, thereby potentially regulating other aspects of cell function such as migration, mechanotransduction and differentiation.

## Results

### Differentiation increases membrane-actin cortex bond strength

**Figure 1:**
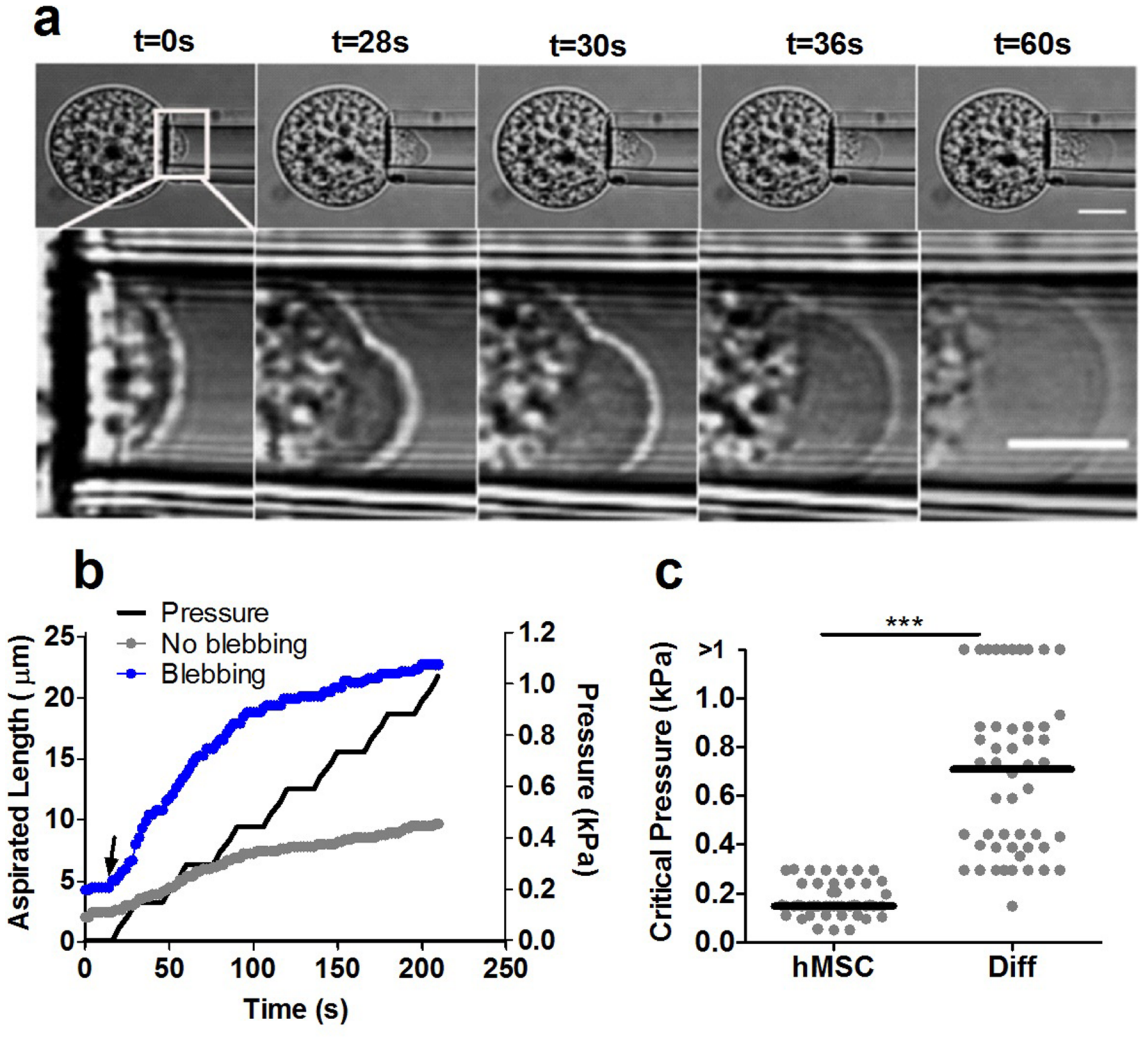
Differentiation strengthens membrane-actin cortex adhesion reducing the susceptibility to blebbing observed in hMSCs. (a) Bright field images of a single representative cell at increasing increments of applied aspiration pressure showing the formation of a membrane bleb at t = 28 s and pressure = 0.15 kPa. Lower images show a magnified view of the leading edge inside the micropipette. Scale bars represent 10 µm. (b) Corresponding data showing the temporal change in aspiration length in response to incremental increases in aspiration pressure. Arrow indicates the onset of a membrane bleb. Data for a non blebbing cell is shown for comparison. (c) Scatter plot showing the critical threshold pressure required for membrane detachment and onset of blebbing for hMSCs and differentiated cells. Cells that did not exhibit bleb formation during testing are classified as having a critical pressure greater than the maximum pressure of 1.0 kPa. Data plotted from two independent experiments with median values indicated by bars, n = 47 cells (hMSC) and 50 cells (Diff). (*** p<0.001, Mann-Whitney U test).

A micropipette aspiration system was used to estimate the critical pressure required for detachment of the membrane and the actin cortex of hMSCs. We examined the effect of chondrogenic differentiation (Diff) induced by TGF-β3, assessed by collagen type-II expression (Supplementary Fig. S1). Individual cells from both groups were placed in suspension and subjected to negative pressure resulting in partial aspiration into the micropipette. The aspiration pressure was applied in a series of seven increments of 1.5 cm H_2_O (0.147 kPa) at a speed of 0.1 cm/s (0.098 kPa/s) allowing 15 s between each increment. The critical aspiration pressure required for membrane-actin detachment and initiation of a membrane bleb was determining from analysis of associated brightfield microscopy images (Fig. 1a). The formation of a membrane bleb resulted in a sudden large increase in aspiration length (Fig. 1b). By contrast, in the absence of blebbing, the aspirated length increased to a lesser extent with each increment of pressure. The pressure at which this bleb initiation occurred and hence the strength of the membrane-cortex adhesion, was significantly lower in hMSCs compared to chondrogenically differentiated cells (Fig. 1c). This shows that hMSCs are more susceptible to membrane blebbing than differentiated cells. In addition we observed that both hMSCs and differentiated cells exhibited membrane blebbing following trypsin mediated detachment from monolayer although this was more prominent in hMSCs compared to differentiated cells. Furthermore, this blebbing behaviour did not lead to cell death but instead blebs were retracted and cells returned to a spherical morphology after approximately 10–15 minutes (Fig. S3).

Previous studies have shown that actin-myosin induced cortical tension can generate sufficient intracellular pressure to induce bleb formation^13^. We therefore examined whether the difference in susceptibility to cell blebbing was associated with differences in cortical tension. There was no statistically significant difference in the cortical tension between differentiated cells and undifferentiated hMSCs (Supplementary Fig. S2). Together, these data suggest that increased membrane-actin cortex bond strength is the main determinant of reduced blebbing in differentiated cells compared to hMSCs.

### Differentiation reduces bleb formation in response to hypo-osmotic challenge

**Figure 2:**
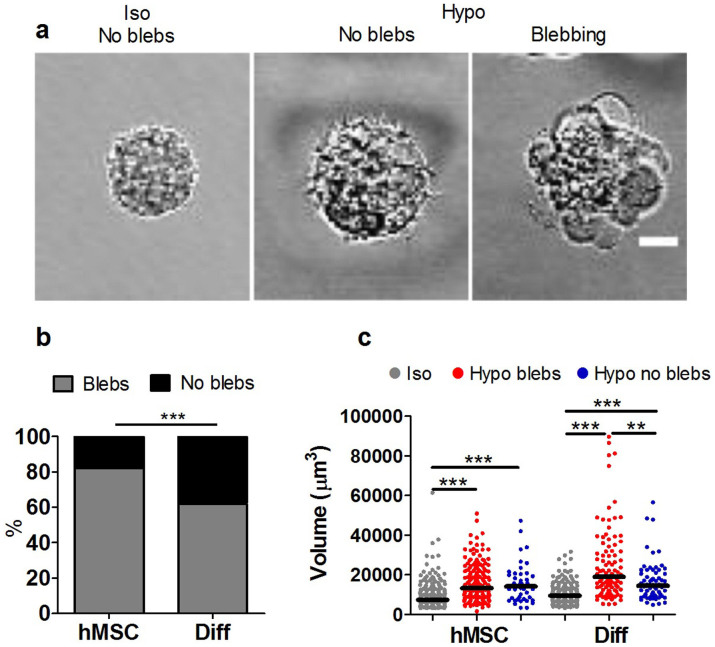
Differentiation reduces membrane blebbing in response to hypo-osmotic pressure and cell swelling. (a) Representative bright field images of hMSCs in suspension in iso-osmotic (330 mOsm/kg) and hypo-osmotic (100 mOsm/kg) media showing the formation of multiple membrane blebs. Scale bar represents 10 µm. (b) The corresponding percentages of cells exhibiting bleb formation in response to hypo-osmotic pressure. (*** p<0.001, Chi-squared test). (c) Scatter plot showing the volume for hMSCs and differentiated cells (Diff) in iso-osmotic conditions and 2–8 minutes after exposure to hypo-osmotic challenge. Data shown separately for blebbing and non-blebbing cells from two independent experiments with median values indicated by bars, (n = 236 hMSC-iso, 173 hMSC-hypo blebs, 53 hMSC-hypo no blebs, 175 Diff-iso, 101 Diff-hypo blebs and 62 Diff-hypo no blebs). *** p<0.001, **p<0.01 Mann Whitney U test.

We next investigated whether the increased susceptibility to membrane bleb formation in hMSCs during aspiration, also occurred in response to intracellular swelling pressure during hypo-osmotic challenge. hMSCs and differentiated cells in suspension were subjected to a sudden reduction in extracellular osmolality (330 to 100 mOsmol/kg). For both cell types this resulted in formation of multiple membrane blebs (Fig. 2a). However, the hMSCs were more susceptible to hypo-osmotic induced membrane blebbing with 82% of cells showing blebs compared to 62% of differentiated cells (Fig. 2b).

Hypo-osmotic challenge was associated with cell swelling such that the cell volume measured after 2–8 minutes was significantly increased compared with corresponding cells in iso-osmotic conditions (Fig. 2c). For differentiated cells, those cells showing blebbing exhibited significantly greater swelling compared to non-blebbing cells. However, there was no significant difference between differentiated cells and hMSCs in terms of the cell volume under iso- or hypo-osmotic conditions. This indicates that the increased blebbing with hypo-osmotic challenge in hMSCs is not due to a difference in swelling.

### Differentiation increases the stiffness of hMSCs

**Figure 3:**
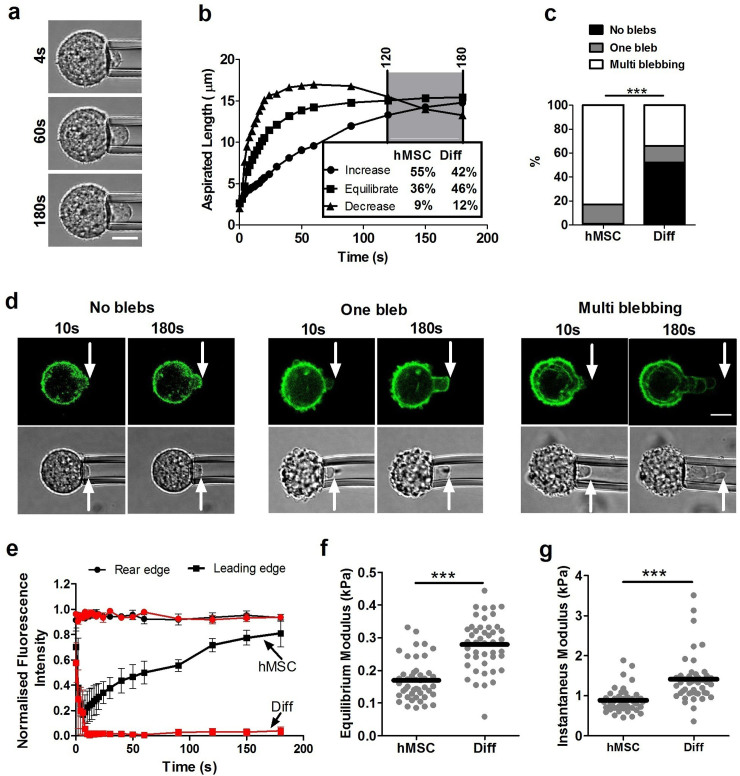
Differentiated cells exhibit increased membrane-actin cortex adhesion and reduced blebbing associated with increased apparent cell stiffness measured by micropipette aspiration. (a) Representative images showing cell aspiration inside the pipette at 4 s, 60 s and 180 s after the application of 0.755 kPa pressure. Scale bar represents 10 µm. (b) Representative plots showing the temporal changes in aspiration length and the three characteristic modes of response depending on the % change in aspirated length from 120 to 180 seconds (see Fig. S4). There were no significant differences between differentiated cells and hMSCs (Chi-squared test; p = 0.249). (c) Histogram showing reduced percentage of cells exhibiting blebbing during aspiration in differentiated cells compared to hMSCs (*** p<0.001 Chi-squared test). Data from four independent experiments, n = 69 cells (hMSC) and 77 cells (Diff). (d) Representative images showing aspirated cells exhibiting either no blebs, one bleb or multi blebbing with visualisation of cortical actin remodelling using LifeAct–TagGFP2. Scale bar represents 10 μm. (e) Plots showing faster actin cortex reformation in the leading edge of a bleb in a hMSC compared to a differentiated cell (Diff). The temporal change in fluorescence intensity at the leading edge is normalised to the initial value at the rear n = 3 cells. Differentiated cells exhibited increased stiffness compared to hMSCs as quantified by significant differences in (f) equilibrium modulus and (g) instantaneous modulus estimated using the SLS model (*** p<0.001, Mann-Whitney U test). Data points plotted from four independent experiments with median values indicated by bars, n = 45 cells in each group (see Table S1).

Micropipette aspiration was used to determine the viscoelastic properties of hMSCs and the effect of chondrogenic differentiation. Individual cells from both groups were placed in suspension and subjected to a step negative pressure resulting in gradual partial aspiration into a micropipette visualised over 180 s (Fig. 3a). Cells exhibited characteristic viscoelastic behaviour in which the aspiration length initially increased rapidly. Cells were characterized into one of three groups: increase, equilibrate or decrease depending on the change in aspiration length over the last 60 seconds of the 180 seconds period (Fig. 3b). A small percentage (8–10%) of both hMSCs and differentiated cells showed retraction with a decrease in aspirated length from 120–180s. This agrees with previous studies of hMSCs^14^. There were no significant differences in the percentage of cells showing each response between hMSCs and differentiated cells (Fig. S4).

In a substantial subpopulation of cells, micropipette aspiration to 7.7 cm H_2_O (0.755 kPa) triggered the formation of one or more membrane blebs inside the micropipette; see Supplementary Movies 1 (no blebbing), Supplementary Movie 2 (one bleb) and Supplementary Movies 3–5 (multi-blebbing). For multi blebbing behaviour some cells has shown three dimensional blebbing. However, in this study we considered multi blebbing as unidirectional. This mechanically induced blebbing was significantly reduced in differentiated cells where 48% of cells exhibited one or more blebs compared to 99% cells in hMSCs (Fig. 3c). To examine the cortical actin remodelling during bleb formation, cells were transduced with LifeAct-TagGFP2. Aspiration of cells into the micropipette resulted in initial distortion of the cortical actin. In the event of bleb formation, the membrane detached from the leading edge of the actin cortex and moved more rapidly inside the pipette (Fig. 3d). In hMSCs, blebbing was followed by reformation of a new actin cortex in the bleb. In differentiated cells, where a bleb did form, the rate of reformation of the actin cortex appeared much slower than in hMSCs (Fig. 3e). We suggest that this is also true in multi blebbing cells although the nature of multi blebbing makes it difficult to visualise actin cortex remodelling (Fig. S5).

Temporal changes in aspiration length were fitted using the standard linear solid model first described by Sato et al^15^ to calculate the equilibrium and instantaneous moduli of both cell types^16^. Sato's model is based on relatively small cell deformation within the micropipette and it does not include blebbing. However, fitting to this model enables the calculation of “effective” elastic properties which are useful for comparison with previous work. The effective equilibrium elastic modulus *E*_∞_ is calculated from equation 1, where Δ*p* is the applied suction pressure, *R_p_* is the pipette radius, *L*_0_ is the initial aspirated length inside the micropipette and *L_s_* is the aspirated length at the end of the experimental observation window (*t* ~ 180*s*); 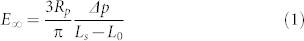
It can be seen in Figures 3f and 3g that differentiated cells have higher equilibrium and instantaneous moduli compared to hMSCs. This correlates with smaller values of *L_s_* in differentiated cells (Supplementary Fig. S6). Differences between the median values for the two types of cells are approximately 0.49 kPa for the instantaneous modulus and 0.14 kPa for the equilibrium modulus.

### Modelling of bleb formation during micropipette aspiration confirms the importance of membrane-cortex bond strength in cell mechanics

Our experimental results show that hMSCs are characterised by a smaller value of the critical pressure for bleb formation (Fig. 1c) and a smaller effective equilibrium elastic modulus (Fig. 3f) than differentiated cells. While these results and other authors' work^4^,^13^,^17^ suggest a link between critical detachment pressure and blebbing, we are far from a complete mechanistic understanding. To get insights into this problem, we have modified the simple one-directional model first proposed by Brugues, where adhesive failure of the membrane from the cortex occurs at a critical pressure dependent on linker protein density and strength^17^. In this model, the time-dependent aspirated length *L* is assumed to satisfy: 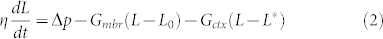
where *t* is time, L* is a reference length, *G_mbr_*(*L*−*L*_0_) is a linear elastic term representing the resistive pressure due to stretching of the membrane, and *G_ctx_*(*L*−L*) is the elastic pressure due to stretching of the cortex attached to the membrane. The pressure drop term 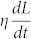 on the left hand side models the viscous dissipation within the cell and the friction with the pipette wall; *η* is a coefficient which is assumed constant. The numerical solution of Eq. (2) proceeds as follows. At the start of the simulation L* is set to the initial aspirated length *L*_0_. In the absence of cortex-membrane detachment, Eq. (2) would give an aspirated length *L* relaxing exponentially to a steady-state value ~Δ*p*/(*G_ctx_* + *G_mbr_*), attained over a time scale *η*/(*G_ctx_* + *G_mbr_*). As *L* increases, however, the resistance opposed by the cortex increases, and this can lead to cortex-membrane detachment. To model this effect, when *G_ctx_*(*L*−L*) = Δ*p_c_*, where Δ*p_c_* is an assigned critical pressure, we set the parameter *G_ctx_*, related to the dilatational modulus of the cortex, to zero. Correspondingly, L* is set to the current aspirated length, to model the fact that new bleb nucleates from the deformed surface of the old bleb. In Brugues's model the resistive elastic pressure is written explicitly in terms of the product of the curvature of the tip of the aspirated portion of the cell within the micropipette and the cortex tension^17^. By contrast, in our model this product is linearised (see Supporting Information), and a single parameter *G_ctx_* is introduced to model the mechanical resistance to cortex stretching. The fact that such a simple model yields *L* − *t* curves that are overall not dissimilar from the experimental ones, and produces dynamics that are dependent on the critical pressure, as in the experiments, suggests that the model assumptions are valid.

**Figure 4:**
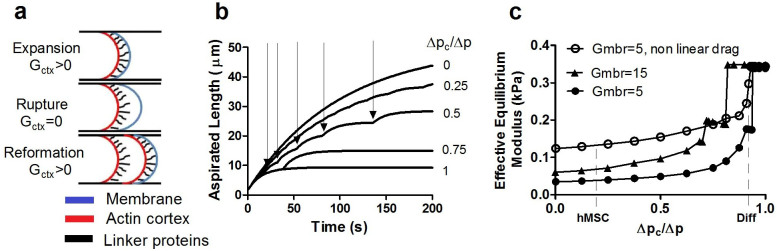
Computational modelling of membrane-actin cortex adhesion and bleb formation during micropipette aspiration confirms that the increased apparent stiffness in differentiated cells is due to increased membrane-actin cortex adhesion. (a) Schematic representation of the hemispherical leading edge of the cell being aspirated within the micropipette. The parameter G_ctx_ models the elastic response of the cortex when attached to the membrane, see equation (2). (b) Aspirated length vs. time curves predicted by Eq. 2 for the same applied pressure used in the experiments of Fig. 3. The curves are for different values of the critical pressure Δp_c_. Slope discontinuities, indicated by arrows for a selected curve, correspond to blebbing events. (c) Effective equilibrium elastic modulus plotted as a function of Δ*p_c_*/Δ*p*, for *G_mbr_* = 5 *Pa*/*μm* and *G_mbr_* = 15 *Pa*/*μm*. For the case *G_mbr_* = 5 *Pa*/*μm* we also report the curve corresponding to non-linear drag defined by a length-dependent friction coefficient *η* = *η*_0_(1 + *L*/*R_p_*) which shows excellent agreement with experimental data for hMSCs and differentiated cells.

The parameters used in the solution of Eq. (2) are estimated from the experiments; details are given in Methods and Supplementary Information. For a given value of *G_ctx_*, the solution of Eq. (2) is controlled by two non-dimensional parameters: the ratio Δ*p_c_*/Δ*p* of the critical pressure to the applied pressure, and the ratio *G_mbr_*/*G_ctx_* of membrane elasticity to cortex elasticity. *L* − *t* curves for Δ*p_c_*/Δ*p* = 0, 0.25, 0.5, 0.75, 1.0 and a fixed value of *G_ctx_*, which has been estimated by fitting to non-blebbing cells, are plotted in Figure 4b. At Δ*p_c_*/Δ*p* = 1.0 (or larger) the failure criterion is not satisfied and no blebbing occurs. For Δ*p_c_*/Δ*p*<1, one or more membrane-cortex detachment events occur, which can be identified as the points where the *L* − *t* curve displays slope discontinuities. In our experiments, such slope discontinuities at bleb initiation cannot be unambiguously identified due to the limited time-resolution, and the fact that cortex-membrane adhesion failure is not a sharp “on-off” event. As the critical pressure Δ*p_c_* decreases, both the number of blebbing events and the value of *L* corresponding to each value of *t* increases, suggesting a more deformable, and thus effectively “softer” cell. To characterise the effective mechanical properties of the cell, we again use the effective equilibrium elastic modulus *E*_∞_ defined in Eq. (1), with *L_s_* now taken as the simulated aspirated length corresponding to the end of the simulation (t = 200s). In Fig. 4c, the effective equilibrium modulus corresponding to the numerical data is plotted vs. Δ*p_c_*/Δ*p* for two values of *G_mbr_*;5 *Pa*/*μm* and 15 *Pa*/*μm*. For *G_mbr_* = 5 *Pa*/*μm*, we also report a curve where we adopt Brugues's approach of taking *η* to be a linear function of *L*, instead of assuming this coefficient to be a constant. Even with constant coefficients, the dependence of the effective equilibrium constant on Δ*p_c_* is strongly non-linear, suggesting that this variable can potentially play a dominant role in modulating the blebbing dynamics of cells. Experimental measurement of the critical pressure for membrane detachment, Δ*p_c_*, provides median values of approximately 0.15 kPa for hMSCs and 0.71 kPa for differentiated cells (Fig. 1c). These correspond to values for Δ*p_c_*/Δ*p* of 0.20 and 0.94 for an applied pressure of 0.75 kPa, respectively. For these experimental values of applied pressure, differentiated cells are thus closer to the range where a strong dependency on pressure is predicted.

### Differentiation reduces cell motility and migration

Bleb formation has been linked to cell migration^7^ and hence we also examined whether changes in blebbing during differentiation were associated with alterations in migration quantified using a scratch assay. The cells were cultured until they reached 90% confluence when a single scratch was made and monitored for 30 hours using brightfield microscopy. The results show that undifferentiated hMSCs migrate faster than differentiated cells (Supplementary Fig. S7). Thus the low membrane-cortex adhesion and inherent blebability of hMSCs compared to differentiated cells is associated with increased migration as well as reduced cell stiffness.

### Differentiation increases F-actin organisation and slows actin dynamics

**Figure 5:**
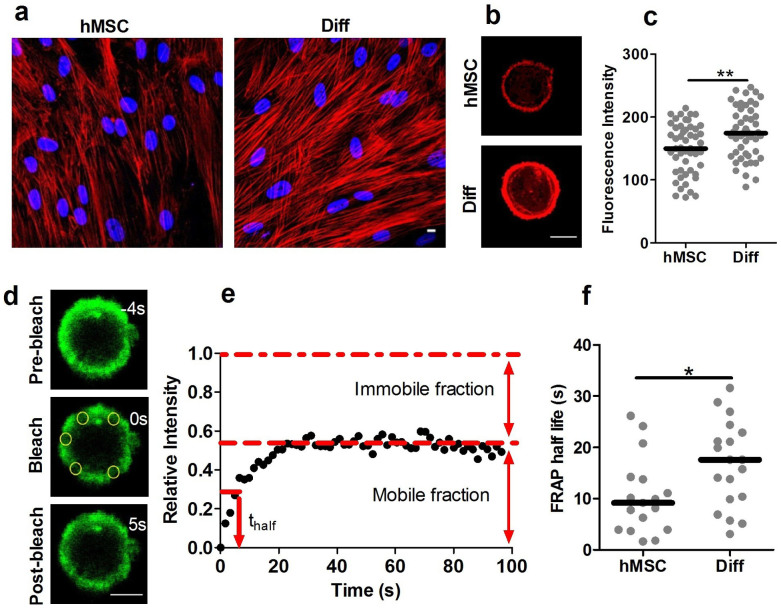
Differentiation increases cortical F-actin expression and slows actin turnover. Confocal images showing increased F-actin organisation in differentiated cells (Diff) compared to hMSCs in (a) monolayer and (b) suspension. F-actin labelled with Alexa555-phalloindin (red). Scale bar represents 10 µm. (c) Scatter plot showing the increased cortical actin staining intensity for differentiated cells in suspension compared to hMSCs (** p<0.01, Mann-Whitney U test). Median values indicated by bars, n = 51 cells (hMSC) and 50 cells (Diff). (d) Analysis of cortical actin dynamics by FRAP of LifeAct-GFP. Representative confocal images pre-bleach and at 0 s and 5 s after photobleaching of five circular regions of interest (ROIs) positioned on the actin cortex (yellow circles). Scale bar represents 10 µm. (e) Temporal changes in mean intensity within the ROIs showing characteristic FRAP behaviour. (f) There was a significant difference in the recovery half life indicating slower remodelling in differentiated cells (Diff) compared to hMSCs (* p<0.05, Mann-Whitney U test). Data plotted from two independent experiments with median values indicated by bars, n = 17 cells (hMSC) and 19 cells (Diff).

The membrane-actin cortex adhesion is likely to be dependant on the density and strength of the ERM linker proteins. This in turn may be influenced by the organisation of the cortical actin. Therefore, we next examined whether chondrogenic differentiation was associated with changes in F-actin organisation quantified from confocal images of cells labelled with AlexaFluor555-phalloidin. Cells induced to undergo chondrogenic differentiation had more pronounced actin stress fibres in monolayer, compared with undifferentiated hMSCs (Fig. 5a). Similarly, differentiated cells in suspension showed increased levels of cortical actin staining (Fig. 5b,c). To examine actin dynamics in the two cell populations we used a customised confocal fluorescence recovery after photobleaching (FRAP) protocol similar to that adopted in previous studies^18^. The cortical actin in both hMSCs and differentiated cells exhibited characteristic FRAP behaviour (Fig. 5d,e) with a mobile fraction of approximately 50% indicative of F-actin turnover also known as treadmilling^19^. The rate of remodelling was slower in differentiated cells as shown by statistically significant differences in the FRAP half-life, t_half _(Fig. 5f). However, there was no difference in the relative mobile fraction (Supplementary Fig. S8). Thus chondrogenic differentiation increases cortical F-actin organisation and reduces turnover rate.

### Higher ERM protein expression in differentiated cells causes the observed changes in blebbing and cell mechanics

**Figure 6:**
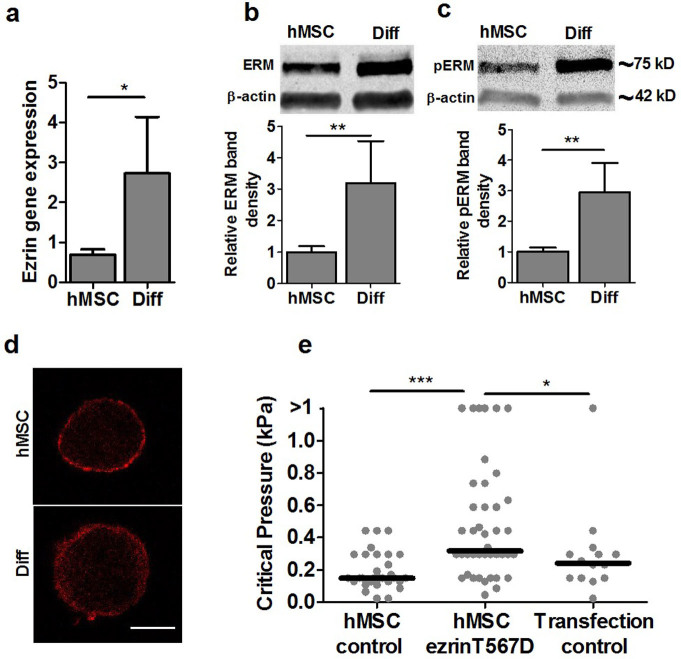
Differentiation up-regulates ERM expression leading to increased membrane-cortex adhesion strength. (a) Real time RT-PCR analysis showing increased ezrin gene expression in differentiated cells compared to hMSCs (* p<0.05, t-test). The level of expression was normalised to GAPDH. Representative cropped western blots showing increased (b) total ERM protein and (c) phosphorylated ERM protein levels in differentiated cells with quantification of immuno-blots including ezrin, radixin and moesin (ERM). Data normalized to β-actin. Full-length blots are presented in supplementary figure S9. (d) Representative confocal images of single cells in suspension showing thicker cortical localisation of ERM (red) in differentiated cells compared to hMSCs. Scale bar represents 10 μm. (e) Scatter plot showing increased critical pressure required for membrane-actin cortex detachment following transfection of hMSCs with ezrin T567D (* p<0.05, *** p<0.001, Mann-Whitney U test). Data taken from two independent experiments with median values indicated by bars, n = 30 (hMSC control), n = 42 (ezrin T567D) and n = 14 (Transfection control).

Previous studies have examined how ERM proteins are involved in cell mechanics and membrane-cortex interactions^20^. We therefore examined ERM gene and protein expression and found that differentiated cells had significantly greater ezrin gene expression compared with hMSCs (Fig. 6a), and an associated increase in total and phosphorylated ERM protein levels (Fig. 6b,c). The distribution of ERM protein in cells visualized using confocal immunofluorescence, showed that in undifferentiated hMSCs, ERM appeared as a thinner cortex localized to the cell membrane in contrast to more widespread appearance in differentiated cells (Fig. 6d). It should be noted that bond strength might be associated not only with the total amount of ERM protein, but also with the extent of ERM phosphorylation which regulates binding to actin filaments.

Finally to confirm whether changes in ezrin expression affect the membrane-actin cortex bond strength, hMSCs were transfected with a plasmid expressing the active form of ezrin T567D, where the N-terminal binds the plasma membrane and the C-terminal binds to F-actin^21^. We performed micropipette aspiration of the transfected cells and found that the critical pressure for membrane detachment and blebbing significantly increased compared to untreated hMSCs or cells subjected to transfection media alone (Fig. 6e). Thus the increased ezrin expression measured in differentiated cells is likely to drive the increase in membrane-cortex adhesion. However up-regulation of ezrin by T567D did not increase the critical pressure to levels measured for differentiated cells (Fig. 1c). This suggests that other factors, such as the increased cortical actin organisation, may also play a role.

## Discussion

In this study we show that the critical pressure for membrane-actin cortex detachment or bond strength is increased following differentiation of human mesenchymal stem cells (hMSCs). We show that this causes hMSCs to be particularly susceptible to membrane blebbing but that this ‘blebability’ is reduced during differentiation. We next examined how alterations in membrane-cortex adhesion influence cell mechanics. During micropipette aspiration, hMSCs and differentiated cells behaved as viscoelastic solid materials, such that the aspirated length reaches an equilibrium state as has been described for other cells types such as endothelial cells^15^ and primary chondrocytes^22^. In our studies using the standard linear solid model, hMSCs were found to have effective equilibrium and instantaneous moduli of 0.15 kPa and 0.8 kPa respectively, in close agreement with previous studies^1^,^23^. We demonstrate that differentiation of hMSCs down a chondrogenic lineage affects cellular viscoelastic properties, characterized by significant increases in instantaneous and equilibrium moduli. We next used a simple mathematical model, based on a uni-directional approximation of the blebbing dynamics, to get insights into the dependence of the effective mechanical parameters of the cell on critical pressure. This model predicts a strong dependence of the effective cell equilibrium modulus, on the critical detachment pressure.

To examine the mechanism responsible for increased bond strength we used quantitative confocal microscopy to measure changes in actin organisation and dynamics. We found that cortical actin in differentiated cells was more developed than in hMSCs, similar to that reported in previous studies^20^. Furthermore, FRAP in live cells transfected with LifeACT-TagGFP2 demonstrated that the differentiated cells have longer recovery times indicative of a more stable actin cortex with slower turnover compared to hMSCs (Fig. 5f). This is in agreement with our observations of slower remodeling within blebs (Fig. 3e). This difference in actin organisation and dynamics may have both a direct effect on cell mechanics by stabilizing the cortex as well as an indirect effect by influencing the bond strength with the cell membrane.

The ERM family of membrane-cortex linker proteins have been shown to regulate stem cell differentiation^12^ as well as cancer metastasis^24^. Here we show that ERM is up-regulated with chondrogenic differentiation at gene and protein level (Fig. 6) and that transfection of hMSCs with the active form of ezrinT567D increases the membrane-cortex bond strength. This is supported by previous studies which report that phosphorylation of ezrin from the inactive form to the active form increases affinity binding to F-actin^21^. ERM has also been shown to influence cell mechanics^25^. However the present study is the first to show that chondrogenic differentiation increases ERM expression leading to increased membrane-cortex adhesion which in turn influences cell mechanics by reducing bleb formation. ERM expression is also linked to bleb-based motility^26^ and thus the observed reduction in cell migration with differentiation may reflect the increased membrane-cortex adhesion and reduced bleb formation. We therefore suggest that stem cells are inherently blebby (high propensity to form blebs) due to low expression of ERM and that this influences characteristic stem cell behavior such as the ability to migrate. Furthermore, the biomechanical changes in membrane-cortex interaction which occur during differentiation of hMSCs are likely to impact on cell mechanics, and other fundamental areas of cell behavior including migration and mechanotransduction.

## Methods

### Cell culture and differentiation

Human bone marrow derived mesenchymal stem cells (hMSCs) were isolated from bone marrow aspirates purchased from a commercial source (Lonza^TM^, Wokingham, UK) as previously described^27^. Cells were seeded at 5000 cells/cm^2^ in 75 cm^2^ flasks and cultured in media consisting of low glucose DMEM, 10% FBS, penicillin (100 U/mL)-streptomycin (100 µg/mL) and 1 ng/mL FGF-2 at 37°C 5% CO_2_. Cells between passages 2 and 6 were used for all experiments. For chondrogenic differentiation cells were plated in 24-well plates at 5000 cells/cm^2^ and cultured for 2 days in hMSC medium to allow cells to attach prior to changing the media to chondrogenic differentiation media consisting of high glucose DMEM, 1% penicillin/streptomycin, 1 mM Sodium pyruvate, 1.5 mg/ml BSA, 40 µg/ml L-proline, (1x) Insulin-Transferrin-Selenium-G supplement, 4.7 µg/ml Linoleic acid, 50 µg/ml L-ascorbic acid, 100 nM Dexamethasone and 10 ng/ml TGF-β3 and culturing for a further 7 days. As a control, cells were maintained in hMSC media throughout the 9 day culture period. In both cases, media was replaced every 2–3 days. For micropipette aspiration and osmotic challenge experiments, cells were detached with 0.25% Trypsin/EDTA for 3–5 minutes, pelleted and suspended in pre-warmed imaging medium (IM) consisting of low glucose DMEM (Pyruvate, no Phenol Red), 1% penicillin/streptomycin, 10% FBS, 4 mM L-Glutamine and 25 mM HEPES for 10 min prior to being transferred to a chamber mounted on an inverted confocal microscope system.

### Micropipette aspiration

Micropipette aspiration was used to determine the critical pressure required for detachment of the cell membrane from the actin cortex and associated membrane blebbing. The micropipette aspiration system is similar to that previously described^28^. A peristaltic pump (MCD standard, ISMATEC) is used to provide precise temporal control over the head of water and hence the aspiration pressure. Micropipettes were made by drawing borosilicate glass capillary tubes (1.0 mm outer diameter and 0.58 mm inner diameter, Narishige, Japan) with a programmable pipette puller (Flaming/Brown micropipette puller, model P-97, Sutter Instruments Co., USA). The micropipettes were fractured on a microforge (MF-900, Narishige, Japan) to obtain an inner diameter of approximately 7–8 µm. The micropipettes were coated with Sigmacote to reduce friction and prevent cell adhesion. The micropipettes were filled with imaging medium (IM) and placed in a holder controlled by a micromanipulator (Patchman NP2, Eppendorf, Germany). A cell suspension prepared in IM was placed in a chamber at room temperature on the inverted stage of a confocal microscope (TCS SP2, Leica Microsystems) with a x63/1.4 NA oil immersion objective lens. An individual cell was subjected to negative pressure in a series of seven increments of 1.5 cm of water (0.147 kPa) every 15 seconds to a maximum pressure of 10.5 cm of water (1.03 kPa). The critical pressure for membrane detachment was taken at the pressure at which a membrane bleb first appeared. Cells that did not bleb were recorded as having a detachment pressure greater than the maximum aspiration pressure for non parametric statistical analysis. The same experimental protocol was also used to calculate the cellular cortical tension as previously described^13^. For full details of the micropipette aspiration system and protocols see supplementary information.

In addition the viscoelastic properties of hMSCs and differentiated cells were determined using micropipette aspiration as described in previous studies^22^,^28^,^29^. Cells were partially aspirated inside a micropipette by applying a step negative suction pressure of 7.7 cm of water (0.755 kPa). A confocal microscope was used to capture brightfield images every 2 seconds over a 180 second period. Temporal changes in cell aspiration length into the micropipette were measured from the images using MatLab and fitted using the well-established standard linear solid (SLS) model to estimate the cellular instantaneous and equilibrium moduli^15^,^30^. All experiments were conducted within 1 hour following cell trypsinisation and detachment from monolayer culture.

### Osmotic challenge for live cells

Bleb formation in undifferentiated hMSCs and chondrogenically differentiated cells was examined in response to sudden hypo-osmotic challenge (100 mOsm/kg). The osmolality of the imaging media was measured at approximately 330 mOsm/kg using a freezing point depression osmometer (3250, Advanced Instruments, Norwood, USA). To adjust the osmolality to 100 mOsm/kg, distilled water was added to the cell suspension which was then gently mixed and placed on a glass slide for imaging. Bright field images of cells were taken for 7 minutes from the point of adjusting osmolality. The percentage of cells with blebs was counted manually from images. The area of each cell was measured using ImageJ and converted into cell volume based on the assumption that the cells are spherical.

### Numerical simulation of bleb dynamics

Equation 2 is solved by an explicit Euler method. The value *G_mbr_* = 5 *Pa*/*μm* was obtained from literature data for the bare membrane tension^13^,^17^. The cortex elasticity (*G_ctx_* = 95 *Pa*/*μm* for the simulations with *G_mbr_* = 5 *Pa*/*μm*) was obtained by fitting to the equilibrium length for non-blebbing cells, which gives an estimate of 100 *Pa*/*μm* for the sum *G_mbr_* + *G_ctx_*. The characteristic value of the friction coefficient (η ≅ 1500 *Pa*·*s*/*μm* was obtained by fitting an exponential to the data for non-blebbing cells. More details are given in the Supporting Information.

### Cell migration assay

hMSCs were seeded at 5000 cells/cm^2^ on 12-well plates and incubated for 2 days where upon the media was changed to induce chondrogenic differentiation or to maintain stem cell phenotype. The cells were cultured in the appropriate media for a further 7 days to confluence. The “scratch” was created with 2 µL pipette tips. Medium was aspirated and cells were washed twice with warm PBS and fresh medium was added to each plate. At 0, 3, 6, 9, 12, 24 and 30 hours images of the wound area were taken to determine the speed of cell migration. ImageJ and a MATLAB script were used to calculate the wound area at different time points.

### Quantification of actin organisation

For quantification of F-actin organisation cells in imaging media were fixed in 4% PFA for 10 min, permeabilised for 5 min in 0.5% Triton X-100/PBS and stained with phalloidin Alexa Fluor555 (25 μl/ml in PBS + 0.1% BSA for 20 min). The cells were then washed with PBS, suspended in distilled water and a drop of stained cells plated on a coverslip and let dry. Cells were mounted with ProLong Gold and imaged using a confocal microscope (Leica SP2) with a 40x oil immersion lens. Confocal section images were made bisecting the centre of individual cells (n = 50 for each group). As in previous studies, the fluorescence intensity of F-actin staining was measured in the cortical region^31^. The mean intensity was calculated for each cell within a beigel region of interest of 1 μm thickness and outside diameter approximately equal to the cell diameter using LAS AF Lite software (Leica, Germany).

For F-actin staining of cells in monolayer, coverslips with adherent cells were fixed with 4% PFA for 10 min, permeabilised with 0.5% Triton X-100/PBS, washed with PBS and stained with phalloidin Alexa Fluor555 (25 μl/ml in PBS + 0.1% BSA for 20 min). Coverslips were then washed with PBS and mounted with ProLong Gold.

### Visualisation of actin dynamics using LifeAct

To image dynamics of cortical actin we used an adenoviral transduction with LifeAct –TagGFP2^32^. Two days prior to the micropipette aspiration or FRAP experiments, cells were transduced by adding adenovirus LifeAct-TagGFP2 F-actin marker at a pre-optimised multiplicity of infection (MOI) according to the manufacture's protocol. For control plates appropriate media without virus was added. After two days incubation (37°C, 5% CO_2_), the virus containing media was removed from the plates and cells were prepared in suspension.

### Fluorescence recovery after photobleaching (FRAP)

Individual cells in suspension expressing LifeAct were attached to the micropipette with a small tare pressure 0.5 cm H_2_O (0.049 kPa) to prevent cell movement. An initial confocal image (488 nm excitation 1% laser power) was obtained bisecting the centre of the cell. This was used to position five circular regions of interest (ROIs, 2 µm diameter) over the cell cortex (Fig. 5d). The FRAP protocol consisted of four pre-bleach images, followed by five bleach images (100% laser power) and then a further sixty consecutive post-bleach images with a dwell time of 1.64 s/image. Quantitative FRAP analysis was performed using the mean fluorescent intensity for the individual ROIs with values normalised to pre-bleach and initial post bleach intensity. A second order exponential equation was fitted to the normalised post-bleach FRAP data to estimate the half-life (t_half_) and the mobile fraction^33^.

### Regulation of ERM expression

Ezrin point mutation construct T567D (constitutively active actin binding and impaired head to tail association) was a kind gift from G. Charras (University College London, UK)^3^. The cDNA was extracted using a High Pure plasmid isolation kit (Roche Diagnostics, Burgess Hill, UK). Prior to transfection, cells were cultured in antibiotic free low glucose DMEM, 10% FBS media overnight. Plasmid transfection was effected using Lipofectamine LTX Plus. For 2 × 10^4^ cells, 0.2 μg of cDNA was used. Cells were cultured for 6 hours in transfection media. Undifferentiated hMSCs were transfected with ezrin T567D followed by replacement with new culturing media. Mechanical testing was performed the following day. Transfection efficiency was approximately 5% for hMSCs, however micropipette aspiration was only performed on successfully transfected cells expressing GFP.

### Statistical Analysis

Statistical analysis was performed using GraphPad Prism 5 software. The t-test was used for real-time PCR and Western blots, and for all other experiments, the non-parametric Mann-Whitney U test was adopted. Statistical significance was reported at p<0.05 (*), p<0.01 (**) and p<0.001 (***) unless otherwise stated.

## Author Contributions

M.K., K.S., L.B. and D.L. designed research, K.S., S.T., L.B. performed research, K.S., S.T., D.L., L.B., M.K. analysed data, K.S., S.T., D.L., L.B., M.K. wrote the paper.

## Supplementary Material

Supplementary Information Supplementary Information

Supplementary Information Supplementary Movie 1

Supplementary Information Supplementary Movie 2

Supplementary Information Supplementary Movie 3

Supplementary Information Supplementary Movie 4

Supplementary Information Supplementary Movie 5
