# Supplementary material for: Stem cell differentiation increases membrane-actin adhesion regulating cell blebability, migration and mechanics
Source: Sci Rep. 2014 Dec 4;4:7307. doi: 10.1038/srep07307 (PMC4255193; doi:10.1038/srep07307)
Supplement: Supplementary Information [file srep07307-s1.pdf]

SUPPLEMENTARY INFORMATION

Stem cell differentiation increases membrane-actin adhesion regulating cell blebability, migration and mechanics

Kristina Sliogeryte[1], Stephen D.Thorpe[1], David A. Lee[1], Lorenzo Botto[1] and Martin M. Knight[1*]

[1]Institute of Bioengineering and School of Engineering and Materials Science, Queen Mary University of London, Mile End Rd, London, E1 4NS, United Kingdom

[*]**Corresponding Author**: Prof Martin Knight, School of Engineering and Materials Science, Queen Mary University of London, Mile End Rd, London, E1 4NS, UK. Tel. +44 (0)20 7882 8868

m.m.knight@qmul.ac.uk

Supplementary Information contains:

Supplementary Table S1

Supplementary Figures S1-S9

Supplementary Movies 1-5

Supplementary Methods

Supplementary References

SUPPLEMENTARY TABLE

Table S1 – Table showing the numbers of hMSC and differentiated cells that were successfully analysed by micropipette aspiration to calculate the instantaneous and equilibrium moduli. Values in parentheses represent the percentage of the total number of cells for which micropipette aspiration was attempted. Cells were rejected from analysis if they did not aspirate or the standard linera solid (SLS) curve fit produced an R^2 value less than 0.95.

Cell type	Total No of cells	No of cells successfully aspirated	No of cells used for model R^2>0.95
hMSC	85 (100%)	69 (81%)	45 (53%)
Diff	82 (100%)	77 (94%)	45 (55%)

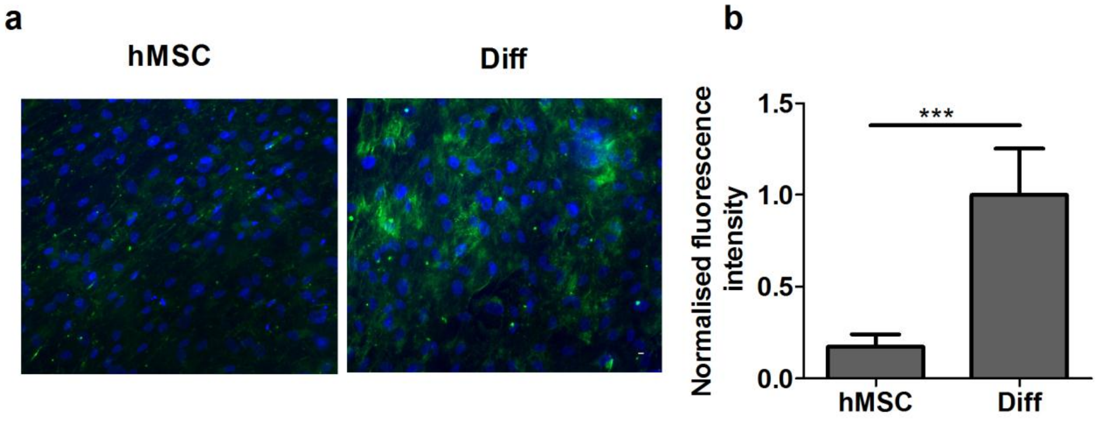

Supplementary Figure S1. Culture of hMSCs in differentiation media (Diff) resulted in increased collagen II expression. (a) Representative confocal images showing increased collagen II immunofluorescence staining in differentiated cells compared to hMSCs. Collagen type II (green), cell nuclear (blue). Scale bar represents 10 µm. **(b)** Histogram showing the increase in fluorescence intensity as measured from confocal images for hMSCs and differentiated cells. Values represent mean with error bars showing standard deviations for 6 fields of view (*** p<0.001, t-test).

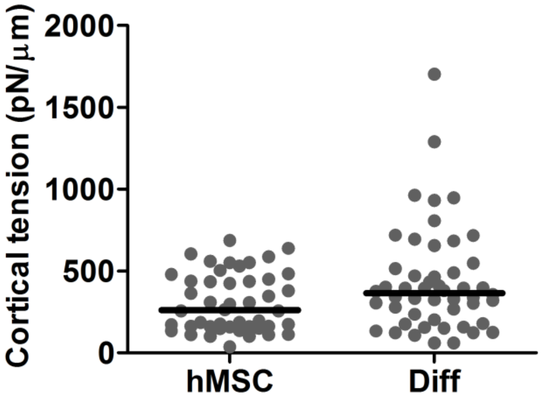

Supplementary Figure S2. Differentiation has no effect on cortical tension. Cells induced toward chondrogenic differentiation (Diff) exhibit no significant difference in cortical tension compared with undifferentiated hMSC cells. Data from two independent experiments, n=46 cells (hMSC) and 50 cells (Diff). (Mann-Whitney U test, p=0.08).

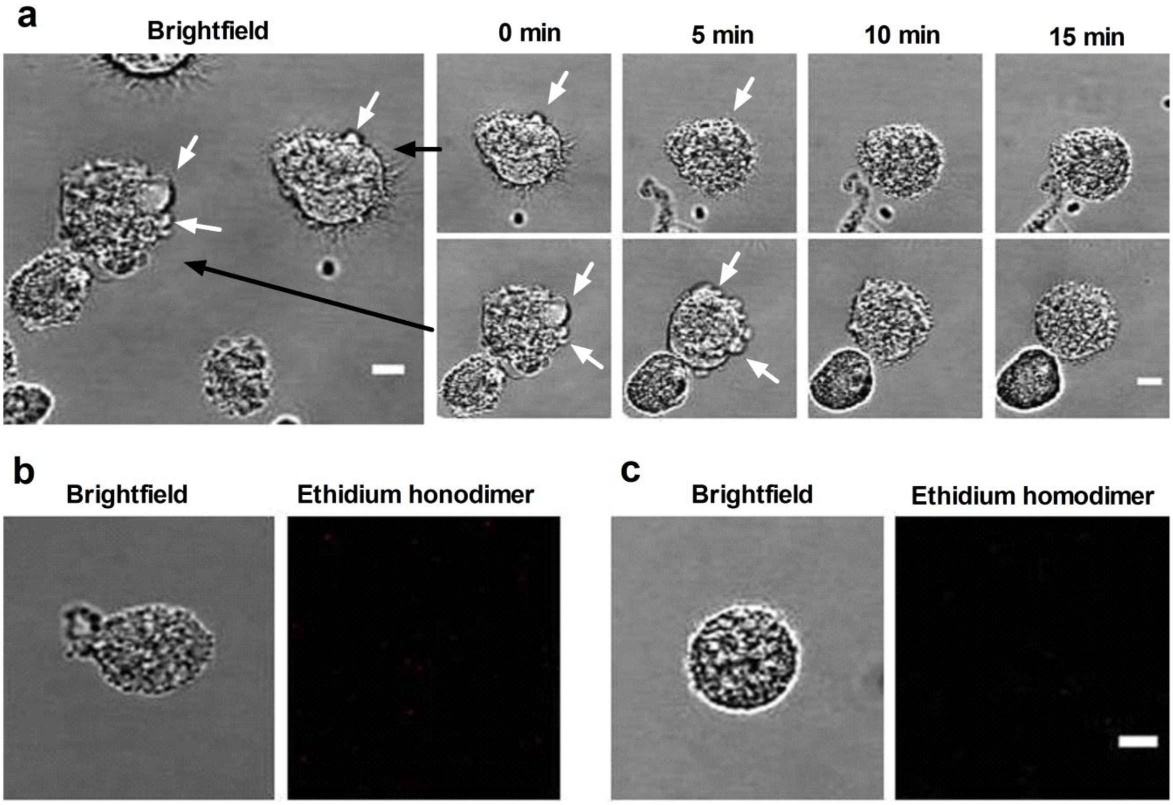

Supplementary Figure S3. hMSCs show extensive blebbing not associated with cell death. (a) Brightfield images of two individual live hMSCs showing extensive blebbing following detachment with trypsin. White arrows indicate membrane blebs. Blebs were fully retracted after 10-15 minutes enabling cells to be subjected to micropipette aspiration. Black arrows indicate the two cells presented in following images. **(b,c)** Blebbing was not associated with cell death or rupture of the membrane as shown by a lack of ethidium homodimer-1 staining. Cells were stained with ethidium homodimer-1 (5 μM) for 15 minutes after detachment and then visualised using confocal microscopy. Scale bar represents 10 μm.

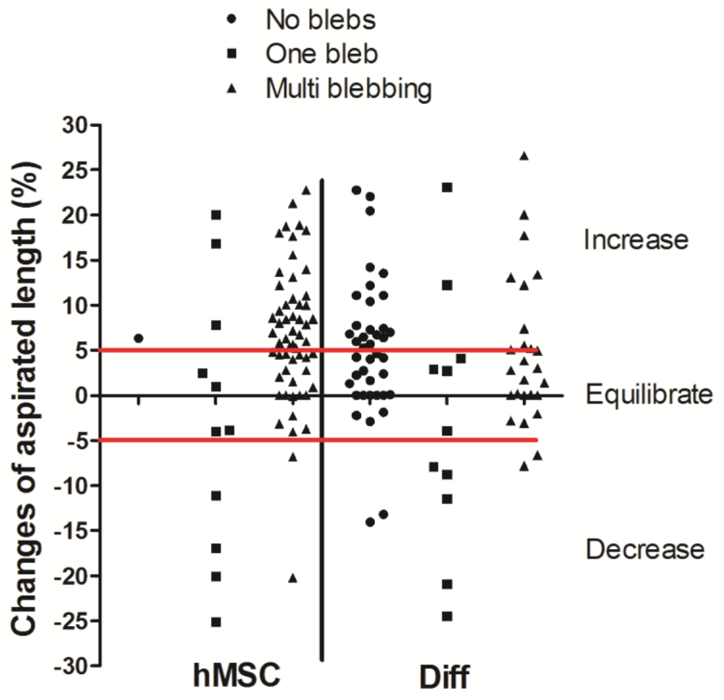

Supplementary Figure S4. Scatter plot showing the change in aspirated length measured from 120-180 seconds after the application of 0.755 kPa aspiration pressure for hMSCs and differentiated cells (Diff). Cells have been classified into three groups: increase, equilibrate and decrease, (see Fig. 3b) based on ±5% thresholds (red lines). Data has shown separately for cells exhibiting no blebs, one bleb or multi blebbing. There were no significant differences between the change in aspirated length or percentage of cells exhibiting each mode of response. Interestingly those cells showing only one bleb were more likely to exhibit a decrease in aspirated length (120-180 seconds) associated with bleb retraction as previously described [1]. Data from four independent experiments, n=69 cells (hMSC) and 77 cells (Diff).

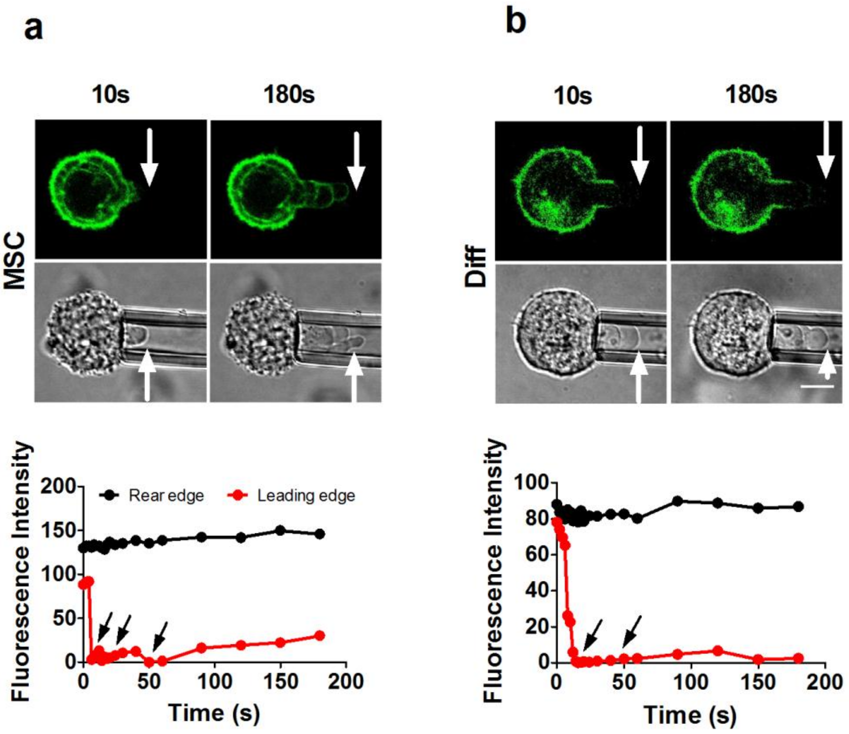

Supplementary Figure S5. Representative fluorescence and brightfield images of (a) an hMSC and (b) a differentiated cell showing multi blebbing behaviour during micropipette aspiration. White arrows show the leading edge of the cell in the micropipette. Scale bar represents 10 µm. Cells were transduced with LifeAct–TagGFP2 for F-actin visualisation. Bottom plots represent temporal fluorescence intensity at the leading and the rear edges. Black arrows indicate the initiation of a new membrane bleb.

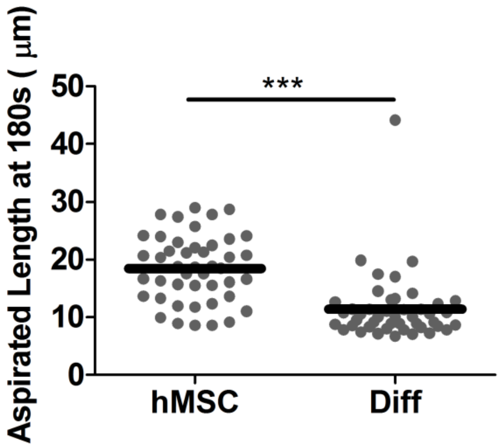

Supplementary Figure S6. Differentiation results in a significant reduction in equilibrium aspiration length indicative of increased cell stiffness. Aspirated length at 180 s is significantly shorter in differentiated cells (Diff) compared to hMSCs. Data from four independent experiments, n=69 cells (hMSC) and 77 cells (Diff). (*** $p<0.001$, Mann-Whitney U test).

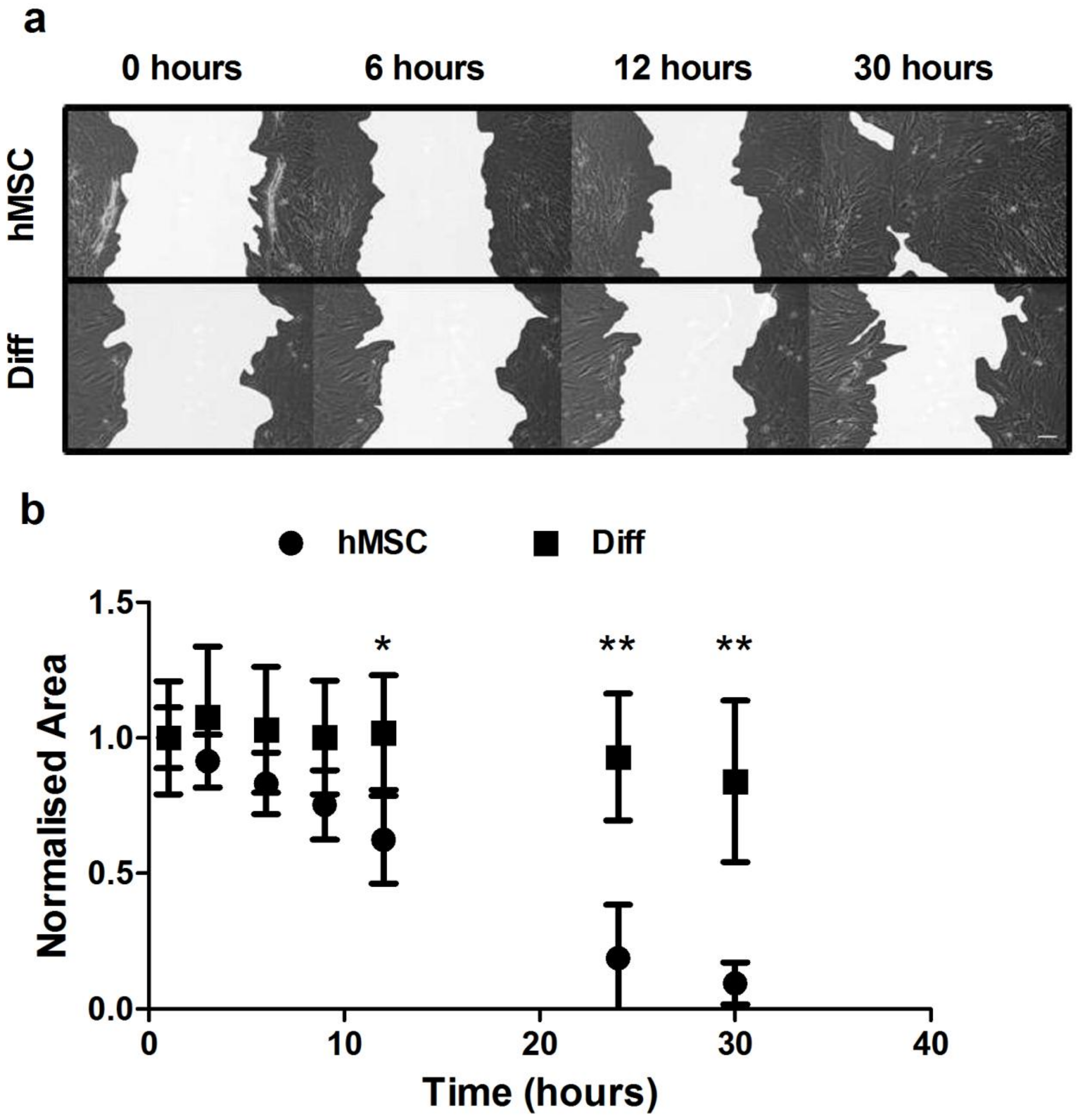

Supplementary Figure S7. Differentiation reduces cell migration. Migration of hMSCs and differentiated cells was assessed based on a scratch assay. **(a)** Bright field microscopy images showing gap closure during cell migration. **(b)** To evaluate the rate of cell migration, the area of gap was calculated at each time increment and normalised to the initial area. Undifferentiated hMSCs exhibit faster migration compared to differentiated cells (Diff) as shown by significantly lower scratch areas at 12, 24 and 30 hours after scratch formation. Data is presented for 5-7 scratch areas for each conditions (* $p<0.05$, ** $p<0.01$, t-test).

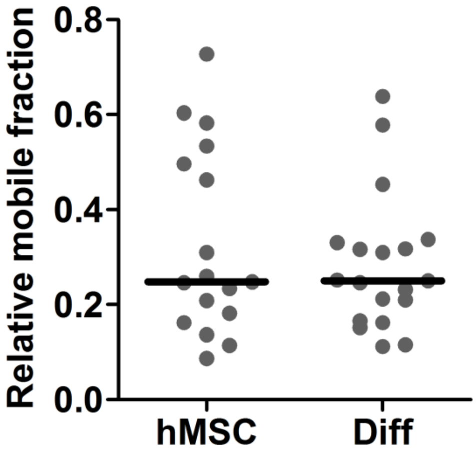

Supplementary Figure S8. Differentiation has no effect on cortical actin mobile fraction based on FRAP analysis of LifeACT-GFP. Data plotted from two independent FRAP experiments with median values indicated by bars, n=17 cells (hMSC) and n=19 cells (Diff). (p=0.76, Mann-Whitney U test.

a

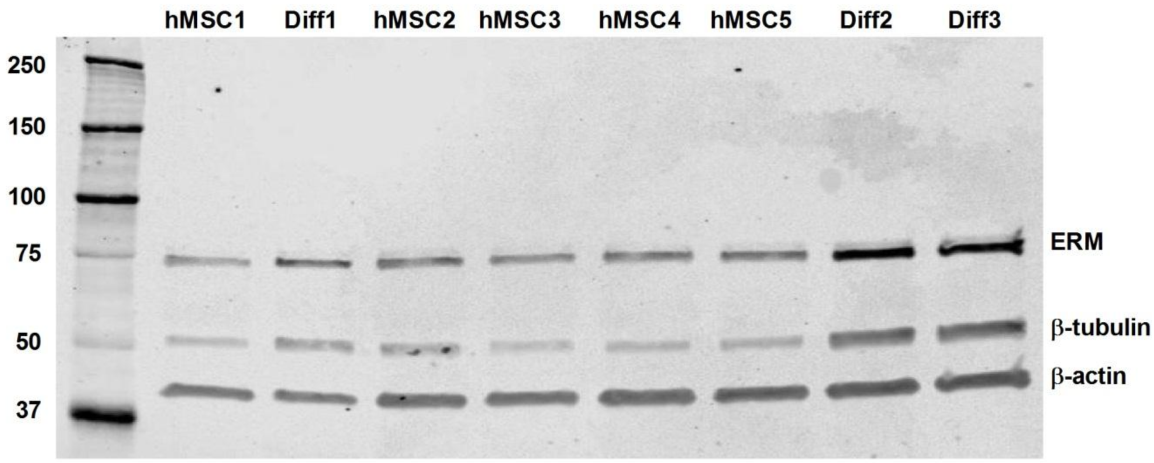

b

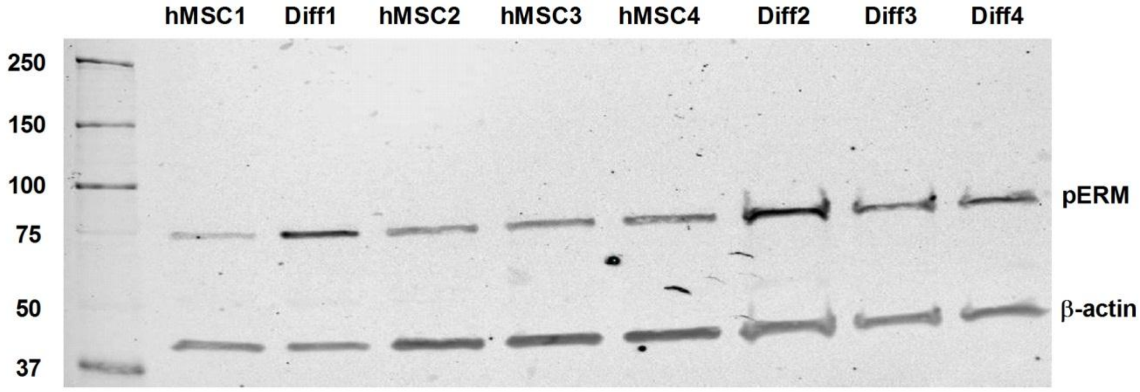

Supplementary Figure S9. Full-length blots corresponding to cropped regions presented in Figure 6. (a) Ezrin/radixin/moesin (ERM) and (b) phosphorylated ezrin/radixin/moesin (pERM). Data presented from 2 replicate experiments (Experiment 1: hMSC1 and Diff1; Experiment 2: remaining samples). 10µg of protein was loaded per well for all samples. Both β-actin and β-tubulin were investigated as control proteins with β-actin used for normalisation as β-tubulin was observed to change with differentiation.

SUPPLEMENTARY MOVIES

The following three movies show a temporal sequence of brightfield microscopy images during micropipette aspiration. The images were collected over 180 s following application of an aspiration pressure of 755 Pa. The micropipettes have an inner diameter of approximately 7-8 µm.

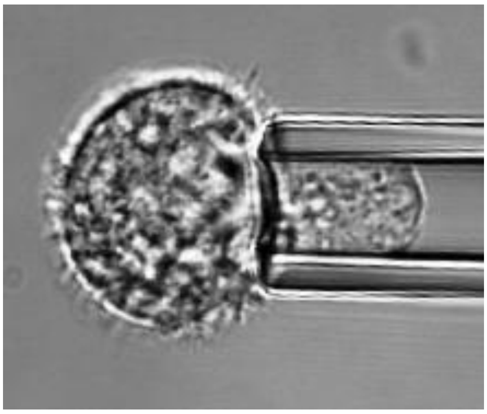

Supplementary Movie 1. Movie showing cell aspiration inside the micropipette without bleb formation. The aspirated portion of the cell moves smoothly inside without membrane-cortex detachment.

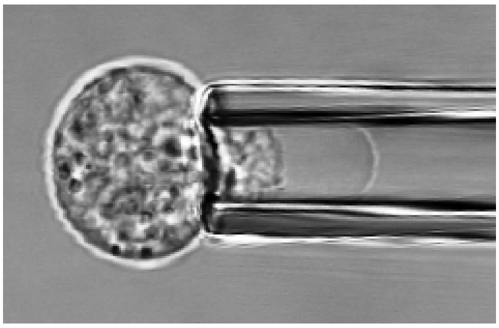

Supplementary Movie 2. Movie showing formation of a single bleb in which the membrane detaches from the cortex allowing rapid bleb expansion.

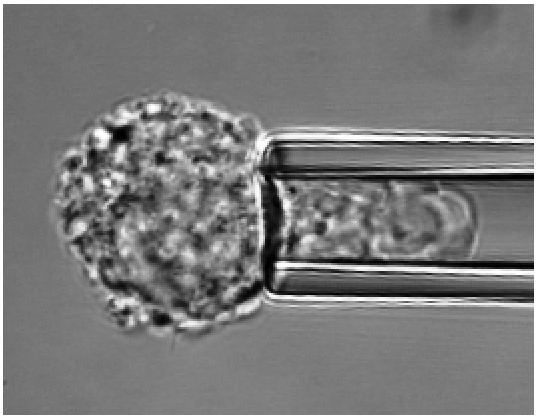

Supplementary Movie 3. Movie showing multi blebbing behaviour in the aspirated region. The first bleb formation is followed by multiple additional blebs leading to continuous multi directional blebbing.

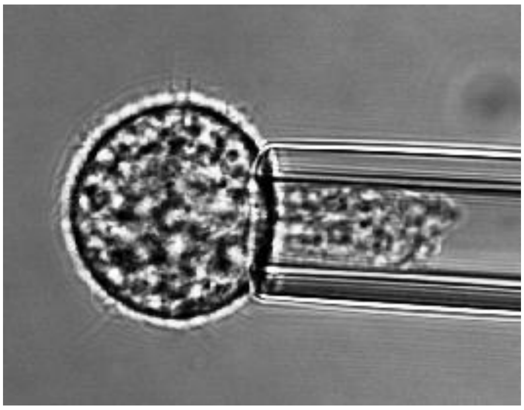

Supplementary Movie 4. Movie showing multi blebbing behaviour in the aspirated region. The first bleb formation is followed by multiple additional blebs leading to unidirectional like blebbing.

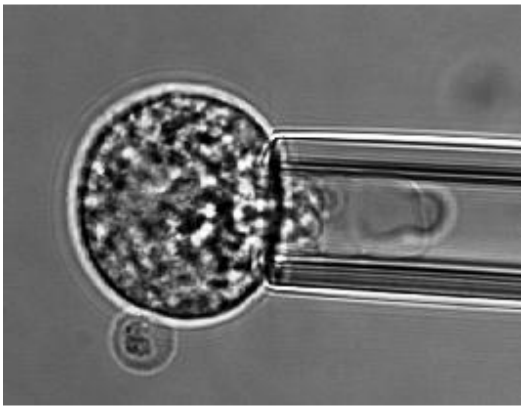

Supplementary Movie 5. Movie showing multi blebbing behaviour in the aspirated region. The first bleb formation is followed by other membrane blebs.

SUPPLEMENTARY METHODS

Chemicals. Dulbecco's Modified Eagle Media (DMEM). Low glucose DMEM, high glucose DMEM, low glucose DMEM (Pyruvate, no L-Glutamine, no Phenol Red), Insulin-Transferrin-Selenium-G supplement 100X, (Gibco, Paisley, UK); foetal bovine serum (FBS), penicillin/streptomycin, sodium pyruvate solution 100mM, bovine serum albumin (BSA), L-Glutamine solution 200mM, HEPES solution 1M, Sigmacote solution, immersion oil, L-proline 40 mg/ml, Linoleic acid-water soluble, L-ascorbic acid, Dexamethasone-water soluble, Trypsin/EDTA, Triton X-100, paraformaldehyde (PFA), Sodium chloride, Sodium dodecyl sulphate (SDS), Trizma base, IGEPAL CA-630 (NP-40), Tween 20, phosphate buffered saline (PBS) (Sigma-Aldrich, Pode, UK); 4X Laemmli buffer (Bio-Rad, Hemel Hempstead, UK); phalloidin (Alexa Fluor555), ProLong Gold (Invitrogen, Paisley, UK); primary antibody rabbit polyclonal anti-collagen type II (Abcam, Cambridge, UK); primary antibody rabbit polyclonal Ezrin/Radixin/Moesin (ERM), primary antibody rabbit monoclonal phosphorylated Ezrin/Radixin/Moesin (pERM) (Cell Signaling Technology, Leiden, Netherlands); primary antibody mouse monoclonal anti-β-tubulin (Sigma Aldrich); secondary antibody goat anti-rabbit (Alexa Fluor488) (Molecular Probes, Paisley, UK); 680RD Goat anti Mouse IgG, 800CW Donkey anti Rabbit IgG (LI-COR Biosciences, Cambridge, UK); recombinant human transforming growth factor beta-3 (TGF-β3) (PromoKine, Heidelberg, Germany); fibroblast growth factor-2 (FGF-2) (PeproTech, London, UK); adenovirus LifeAct-TagGFP2 F-actin marker (Ibidi, Germany); Sodium deoxycholate (Alfa Aesar, Lancashire, UK).

Collagen II immunohistochemistry. Collagen type II expression was identified as a marker of chondrogenic differentiation using immunohistochemistry. Cells cultured on coverslips were rinsed in PBS and blocked with goat serum. Cells were incubated for 1 hour at room temperature with the primary antibody, rabbit polyclonal anti-collagen type II antibody (1:200), then washed with PBS and incubated in the secondary antibody, Alexa Fluor 488 conjugated goat anti-rabbit IgG antibody (1:1000) for 1 hour. Coverslips were finally rinsed in PBS and mounted with ProLong Gold for confocal microscopy.

Measurement of instantaneous and equilibrium moduli using micropipette aspiration. A step negative pressure of 7.7 cm of water (755 Pa) was applied in 2 seconds (3.85 cm/s) and held for 180 seconds. Temporal changes in aspiration length, measured every 2 seconds, were fitted in MATLAB (Cambridge, UK) using the standard linear solid (SLS) model which represents the cell as a homogeneous, incompressible solid with a poisons ratio of 0.5 [2,3]. The model can be represented by a

linear spring with elastic constant k_1 arranged in parallel with a dashpot with viscosity μ and a second elastic spring with elastic constant k_2. When a negative step pressure is applied, the aspiration length of the cell inside the micropipette (L) may be described as a function of time (t) using the following equation (S1).

$$L(t) = \frac{\Phi(\eta) a \Delta p}{\pi E} \times \left[1 + \left(\frac{k_1}{k_1+k_2} - 1\right) \exp\left(-\frac{t}{\tau}\right)\right] \quad \text{(S1)}$$

The applied step pressure is given by ΔP, E is the Young's modulus, a and b are the inner and outer radii of the micropipette respectively, τ is the exponential time constant and $\varphi(\eta)$ is a wall function which depends on the wall parameter η as given in equation (S2). In a wide range of experiments the function can be assumed to be 2.0 1 [2].

$$\eta = \frac{(b-a)}{a} \quad \text{(S2)}$$

Cells were excluded from analysis if they were completely aspirated, did not aspirate at all or the model curve fit R^2 value was less than 0.95. Table S1 indicates the numbers of cells analysed based on the above rejection criteria. The instantaneous modulus (E_0) and the equilibrium modulus (E_∞) are based on the elastic constants k_1 and k_2 as shown in equation (S3).

$$E_0 = \frac{3}{2}(k_1 + k_2), \quad E_\infty = \frac{3}{2} k_1 \quad \text{(S3)}$$

Measurement of Cortical Tension using micropipette aspiration. For measurement of cortical tension, aspiration pressure was applied in a series of increments. The critical threshold pressure ΔP_c was determined at the point where the aspirated portion of the cell formed a hemispherical protrusion such that the aspirated length, L, equalled the inner micropipette radius, a. The cortical tension, T, was then calculated using the following equation based on the liquid drop model, where R_c is the radius of the cell outside of the micropipette equation (S4) [4].

$$T = \frac{\Delta P_c}{2\left(\frac{1}{a} - \frac{1}{R_c}\right)} \quad \text{(S4)}$$

Viscoelastic model of time-dependent bleb formation during micropipette aspiration. The mathematical model in equation (2) is a dynamic version of the widely used static "liquid-drop" model for micropipette aspiration experiments [5], amended to account for the pressure drop due to the motion of the fluid within the cell and wall friction. The right-hand side of equaton (2) can be recognised as the Laplace pressure $\cong 2(\gamma_{mbr}/R_{mbr} + \gamma_{ctx}/R_{ctx})$ acting across the composite interface made by the bare membrane plus the cortex; R_{mbr} and R_{cortex} are the radii of curvature of membrane and cortex, respectively [4,6], and γ_{mbr} and γ_{ctx} are the corresponding surface tensions. In the linear regime, $\gamma_{mbr} = k_{mbr}\alpha$ and $\gamma_{ctx} = k_{ctx}\alpha$, where $\alpha \cong \dfrac{R_p(L - L_{ref})}{2R_c^2}$ is the areal strain, and k_{mbr} and k_{ctx} are the area compressibility moduli of membrane and cortex, respectively; L_{ref} is the aspirated length in the reference (unstrained) configuration, $R_p \cong 3.5\mu m$ is the inner pipette radius, and $R_c \cong 12.5\mu m$ is the radius of curvature of the portion of the cell outside of the pipette. Since R_{mbr} and R_{ctx} are approximately equal to R_p, we can write

$$G_{mbr} \cong k_{mbr} / R_c^2 \qquad \text{(S5)}$$

and a similar equation for G_{ctx}. For a bare membrane, $k_{mbr} \cong 0.8 mN/m$ [6], thus $G_{mbr} \cong 5Pa/\mu m$. For our experiment, the corresponding membrane tension when $L - L_0 = R_p$ is $\gamma_{mbr} \cong GR_p^2/2 \cong 30 pN/\mu m$, comparable to the value $40 pN/\mu m$ reported by Tinevez *et al* [4]. The sum $G_{ctx} + G_{mbr}$, and from this the elastic constant of the cortex, can be estimated from the average equilibrium length observed for non-blebbing cells, $L_{eq} \cong 9\mu m$. Using the stationary solution of equation (2) we obtain $G_{cortex} + G_{mbr} = \Delta p/(L_{eq} - L_0) \cong 100 Pa/\mu m$. With $G_{mbr} \cong 5Pa/\mu m$, this gives $G_{ctx} \cong 95 Pa/\mu m$ The elastic constant of the cortex is thus much larger than that of the bare membrane.

The viscous pressure drop is taken to be proportional to the aspirated length velocity dL/dt via a friction coefficient η. It is not clear whether η should be taken as a constant [7] or a function of L [6]. While the length of the protrusion within the pipette increases, which would suggest a monotonically increasing dependence of η on L, most of the dissipation is expected to occur near the hemispherical tip of the aspirated portion of the cell. The latter observation would suggest a constant value of η (at least asymptotically). In our simulations, we have resorted to initially assume a constant friction coefficient $\eta = \eta_0$ see (Fig. 4 b,c), and later evaluate the effect of replacing this constant with the linear relation $\eta = \eta_0(1 + L/R_p)$ suggested by Brugues et al. [6] (Fig. 4c). Fitting the dynamics of non-

blebbing cells to an exponential with a single relaxation time gives $\eta_0 \cong 1500 Pa \cdot s/\mu m$. Note that the effective friction coefficient is known to depend strongly on the ratio between the pipette and cell radii [7], and may also be a strong function of the cell rigidity, since a stiffer cell should produce a larger friction with the wall.

The effective equilibrium modulus given by the simulation varies between that of the bare membrane for $\Delta p_c/\Delta p$ = 0 to that of the compound actin cortex-membrane for $\Delta p_c/\Delta p$ = 1. For relatively small values of $\Delta p_c/\Delta p$ the dependence of the equilibrium elastic modulus on $\Delta p_c/\Delta p$ is relatively weak, but it becomes considerably stronger as $\Delta p_c/\Delta p$ approaches 1. The range of critical pressures for which such dependence is strongest depends sensitively on the value of G_{mbr}, with larger effective elastic constants for smaller values of Δp_c associated to larger values of G_{mbr}. The dependence of the effective equilibrium modulus on Δp_c predicted by the model with a length dependent friction coefficient is somewhat weaker than that predicted by a constant friction coefficient, but the overall trend is qualitatively similar.

Both Brugues's model and ours rely essentially on the same fundamental assumption. Inevitably there are strong working hypotheses in the model, including the assumption of unidirectional propagation of the blebs, the assumption that all blebs are identical and are regulated by the same failure criterion, and the absence in the model of time-scales of cortex reformation (in Fig.3d we see for instance that the actin concentration at the tip of the aspirated portion of the cell tends to decrease somewhat in the later stages of micropipette aspiration).

Fluorescence recovery after photobleaching (FRAP). Cells transduced with LifeAct-TagGFP2 were suspended in imaging media and imaged on a Leica SP2 confocal microscope equipped with a X63/1.4 NA oil immersion objective. For the FRAP experiments cells were held using the micropipette aspiration system with a tare pressure of 0.5 cm of water (49.05 Pa). A single confocal section was made bisecting the centre of the cell. This image was then used to position five circular regions of interest (ROI) 2 µm in diameter placed on the cell cortex as shown in figure 5D. The bleaching within the circular ROIs was performed by using the 488 nm Argon beam at 100% laser power. All other images were conducted at 1% laser power. The FRAP protocol consisted of 4 pre-bleach images, 5 bleach images followed by 60 post-bleach images. The dwell time for each image was 1.64 seconds and all images were conducted sequentially. Pre- and post-bleach images were obtained at a 512 x 512 format yielding a pixel size of 0.15 x 0.15 µm. Cells were maintained at room temperature during all experiments and time limited to 1 hour after trypsinization. The FRAP analysis

was performed using an easy FRAP tool implemented using MATLAB [8]. For analysis we measured the fluorescence intensity of 3 regions of interest (ROI1, ROI2 and ROI3) at the correspondent time points. The fluorescence intensity of pre-bleach area $I(t)_{ROI1}$, the total fluorescence intensity of all cell $I(t)_{ROI2}$ and background fluorescence intensity $I(t)_{ROI3}$. Before data analysis, the background intensity is subtracted from all measurements equations (S6 and S7):

$$I'(t)_{ROI1} = I(t)_{ROI1} - I(t)_{ROI3} \quad (S6)$$

$$I'(t)_{ROI2} = I(t)_{ROI2} - I(t)_{ROI3} \quad (S7)$$

We applied a double normalization method [9] for the intensity analysis to correct the acquisition bleaching effect or fluctuation in laser intensity. The average pre-bleach intensity of the whole cell is divided by the intensity of the whole cell at each time point and multiplied by the FRAP region intensity at that time point equation (S8).

$$I''(t) = \left[\frac{\frac{1}{n_{pre}}\sum_{t=1}^{n_{pre}} I'(t)_{ROI2}}{I')(t)_{ROI2}}\right] \times \left[\frac{I'(t)_{ROI1}}{\frac{1}{n_{pre}}\sum_{t=1}^{n_{pre}} I'(t)_{ROI1}}\right] \quad (S8)$$

Temporal changes in the normalised intensity were fitted with a double term exponential equation yielding values for the half-life t_{half}.

Quantitative real-time PCR. The total RNA was extracted from undifferentiated hMSCs and hMSCs induced to undergo chondrogenic differentiation. Cells were cultured in 6 well plates under control and chondrogenic differentiation conditions, then lysed in buffer containing β-mercaptoethanol and scraped to remove cells from the plate surface. The RNA was isolated according to manufacturer's instruction using a RNeasy Plus Mini Kit (Qiagen, Manchester, UK). RNA samples were quantified using the NanoDrop ND-100 spectrophotometer (Thermo Scientific, Waltham, USA) and quality assessed on 2% agarose MOPS formaldehyde gel. RNA was reverse transcribed to cDNA using the Qiagen Quantitect cDNA synthesis kit (Qiagen, Manchester, UK). Primer/probe sets were designed and purchased from (Sigma-Aldrich). The cDNA was amplified using a DNA thermal cycler (Mx3000P, Agilent Technologies, Santa Clara, USA) in a 10 µl reaction volume containing required concentrations of PCR reagents. The amplification was performed under the following conditions. The reaction was denatured at 95°C for 15 min, followed by 40 cycles amplification (denaturation at 95°C for 3 s; annealing 60°C for 30 s and extension at 95°C for 30 s). Human glyceraldehyde-3-phosphate dehydrogenase (GAPDH) was chosen as a control gene. A serial dilution of known cDNA was used for to establish a standard curve. The ΔCt for specific gene expression was determined relative to Ct of

GAPDH. The primer for ezrin linker protein was used [10] as follows: 5’-AGCGCATCACTGAGGCAGAG-3’ (forward), 5’-GCCGCAGCGTCTTGTACTTG-3’ (reverse). Sequences of ezrin, radixin and moesin are very similar so this primer is likely to detect changes in other ERM protein genes.

Western blotting. Protein isolation and quantity was measured using standard methods [11]. Cells were cultured and differentiated for 7 days as explained above. Cells then were washed with chilled PBS and lysed in RIPA (Radio Immuno Precipitation Assay) buffer (150 mM sodium chloride, 1% NP-40, 0.5% sodium deoxycholate, 0.1% SDS and 50 mM Tris-HCl) containing 200 mM activated Na_3VO_4 and protease inhibitors (Complete mini, Roche) and lysed on ice for 15 min. This was followed by repetitive disruption of lysate through a 21 gauge needle and centrifugation at 12000g for 15 minutes at 4°C. The protein concentration in supernatant was determined using a BCA protein assay kit (Fisher Scientific, UK). Cell lysates of 10 µg were mixed with Laemmli buffer and denatured by heating at 100°C for 5 min. Samples then were loaded into a Mini-Protean TGX Precast Gel (Bio-Rad, UK), and proteins were transferred to Mini Nitrocellulose Transfer membranes (Bio-Rad, UK). The membranes were blocked with 5% of non-fat milk in Tris-buffered saline (TBS) with 0.1% Tween and then were incubated overnight with primary antibodies (1:1000 dilution for ERM and pERM, and 1:5000 for β-actin). β-actin was used as a control. After being washed with TBS+0.1% Tween and incubated for 1 hour with secondary antibodies (1:10000 dilutions for 680RD Goat anti-Mouse IgG and 800CW Donkey anti-Rabbit IgG). Membranes were washed again in TBS+0.1% Tween and prepared for image processing using an Odyssey infrared Imaging system (LI-COR).

SUPPLEMENTARY REFERENCES

1 Tan, S. C. *et al.* Viscoelastic behaviour of human mesenchymal stem cells. *BMC Cell Biol* **9**, 40, (2008).

2 Theret, D. P., Levesque, M. J., Sato, M., Nerem, R. M. & Wheeler, L. T. The application of a homogeneous half-space model in the analysis of endothelial cell micropipette measurements. *J Biomech Eng* **110**, 190-199, (1988).

3 Sato, M., Theret, D. P., Wheeler, L. T., Ohshima, N. & Nerem, R. M. Application of the micropipette technique to the measurement of cultured porcine aortic endothelial cell viscoelastic properties. *J Biomech Eng* **112**, 263-268, (1990).

4 Tinevez, J. Y. *et al.* Role of cortical tension in bleb growth. *Proc Natl Acad Sci U S A* **106**, 18581-18586, (2009).

5 Hochmuth, R. M. Micropipette aspiration of living cells. *J Biomech* **33**, 15-22, (2000).

6 Brugues, J. *et al.* Dynamical organization of the cytoskeletal cortex probed by micropipette aspiration. *Proc Natl Acad Sci U S A* **107**, 15415-15420, (2010).

7 Evans, E. & Yeung, A. Apparent viscosity and cortical tension of blood granulocytes determined by micropipet aspiration. *Biophys J* **56**, 151-160, (1989).

8 Rapsomaniki, M. A. *et al.* easyFRAP: an interactive, easy-to-use tool for qualitative and quantitative analysis of FRAP data. *Bioinformatics* **28**, 1800-1801, (2012).

9 Phair, R. D., Gorski, S. A. & Misteli, T. Measurement of dynamic protein binding to chromatin in vivo, using photobleaching microscopy. *Method enzymol* **375**, 393-414, (2003).

10 Jiang, Q. Y. *et al.* RNAi-mediated blocking of ezrin reduces migration of ectopic endometrial cells in endometriosis. *Mol Hum Reprod* **18**, 435-441, (2012).

11 Chen, Q. Y. *et al.* Silence of ezrin modifies migration and actin cytoskeleton rearrangements and enhances chemosensitivity of lung cancer cells in vitro. *Mol Cell Biochem* **377**, 207-218, (2013).
